# Supplementary material for: Telomeres in ICF syndrome cells are vulnerable to DNA damage due to elevated DNA:RNA hybrids
Source: Nat Commun. 2017 Jan 24;8:14015. doi: 10.1038/ncomms14015 (PMC5286223; doi:10.1038/ncomms14015)
Supplement: Supplementary Information — Supplementary Figures, Supplementary Tables, and Supplementary References [file ncomms14015-s1.pdf]

Supplementary Figure 1

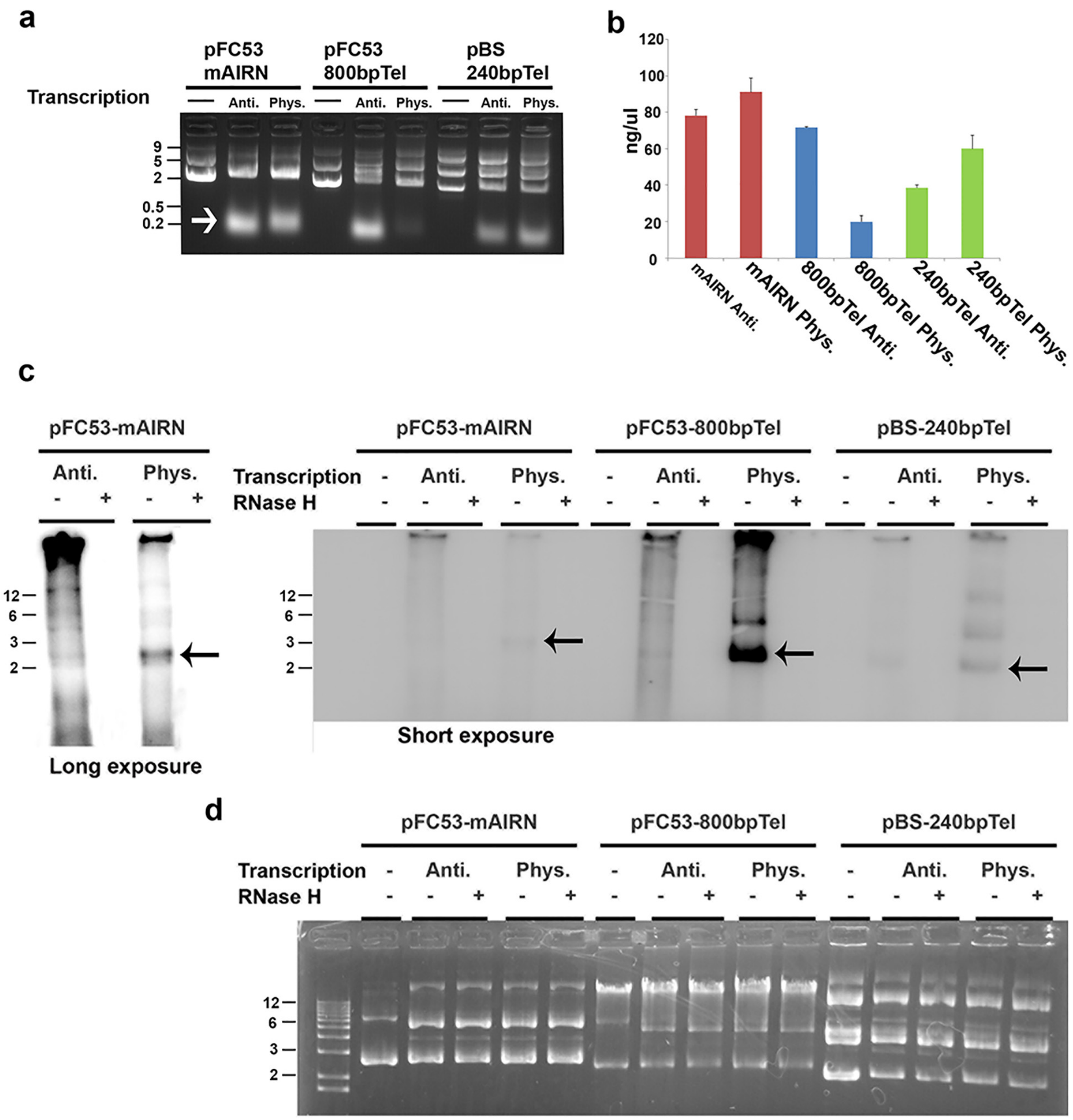

DNA:RNA hybrids form *in vitro* between telomeric DNA and TERRA

Two plasmids containing human telomeric repeats $(TTAGGG)_n$ were utilized for this experiment: a pFC53 plasmid containing 800bp of telomeric repeats (pFC53-800bpTel), and a pBS plasmid containing 240bp of telomeric repeats (pBS-240bpTel). A pFC53 plasmid containing a positive control for formation of DNA:RNA hybrids, mAIRN, was used as well (pFC53-mAIRN)[1]. (**a&b**) Each of the plasmids was transcribed both in the physiological direction of transcription (Phys.) and the reverse, anti-physiological orientation (Anti.). In order to exclude the possibility that increased transcription from one of the two orientations would influence the degree of hybrid formation, we both

visualized the RNA products by gel electrophoresis (a white arrow points to the position of RNA transcribed *in vitro*) (**a**), and quantified the RNA yields generated from both orientations by a Qubit fluorometric quantitation assay (ThermoFisher Scientific), following the manufacturer's protocol. Size markers in kb appear on the left. (**b**). The RNA levels generated from both orientations did not differ substantially in the case of the pFC53-mAIRN and pBS-240bpTel plasmids. In the case of the pFC53-800bpTel plasmid, transcription was markedly lower from the physiological orientation, similar to what had been reported previously with a similar plasmid containing an 800bp fragment of telomeric repeats[2]. (**c&d**) After validating that no excessive transcription occurs in the physiological orientation, each plasmid was transcribed in the presence of ^{32}P-UTP, both in the physiological direction of transcription (Phys.) and the reverse, anti-physiological orientation (Anti.). Each transcribed sample was split equally into two aliquots that were either treated (+) or untreated (-) with bacterial RNase H (M0297, *New England BioLabs*), an endonuclease that specifically degrades RNA present in DNA:RNA hybrids. Samples were run on an agarose gel. DNA:RNA hybrids are visualized as labeled migrating species that disappear following treatment with RNase H. Size markers in kb appear on the left. A photograph of the gel after staining with ethidium bromide appears in (**d**), while (**c**) corresponds to the same gel after drying and exposure to a phosphorimager screen. A clear signal was obtained from the telomeric repeat-containing plasmids only when transcription was carried out in the physiological direction (black arrows), and this signal disappeared following RNase H treatment. The signals arising from the telomeric plasmids were stronger than the signal arising from the control region, shown previously to form DNA:RNA hybrids[1]. A longer exposure of the control samples is shown on the left in (**c**). Note that the generation of DNA:RNA hybrids was the most efficient in the case of the pFC53-800bpTel plasmid, even though the transcription in the physiological orientation was markedly lower (**a&b**).

Supplementary Figure 2

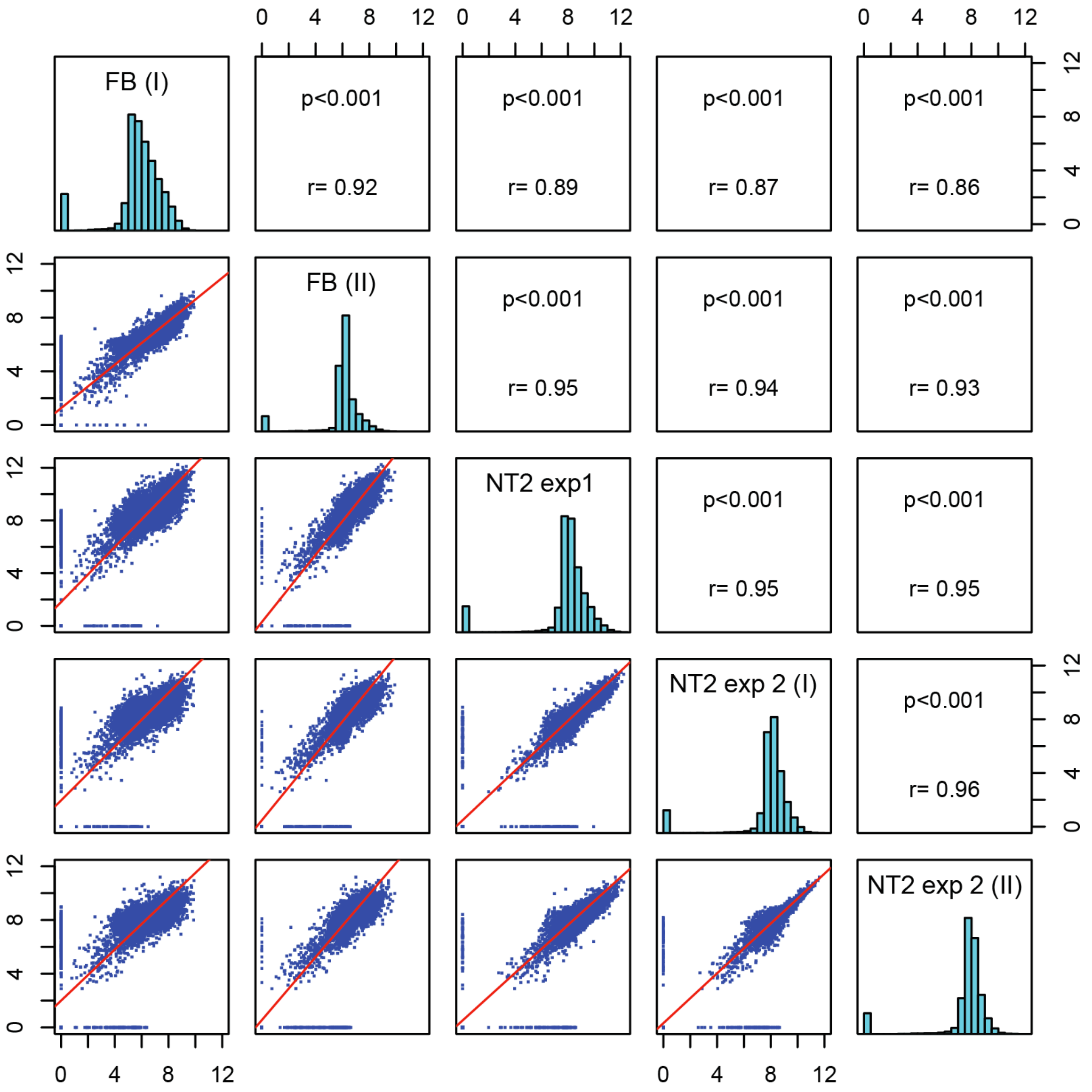

Systemic correlation analysis of DRIP-seq values across independent experiments
XY correlation plots of DRIP-seq signal showing high reproducibility between cell lines and replicates. Histogram of corresponding signal, p-value, and Pearson coefficient are indicated. All DRIP-seq signals are in $\log_2$ scale.

Supplementary Figure 3

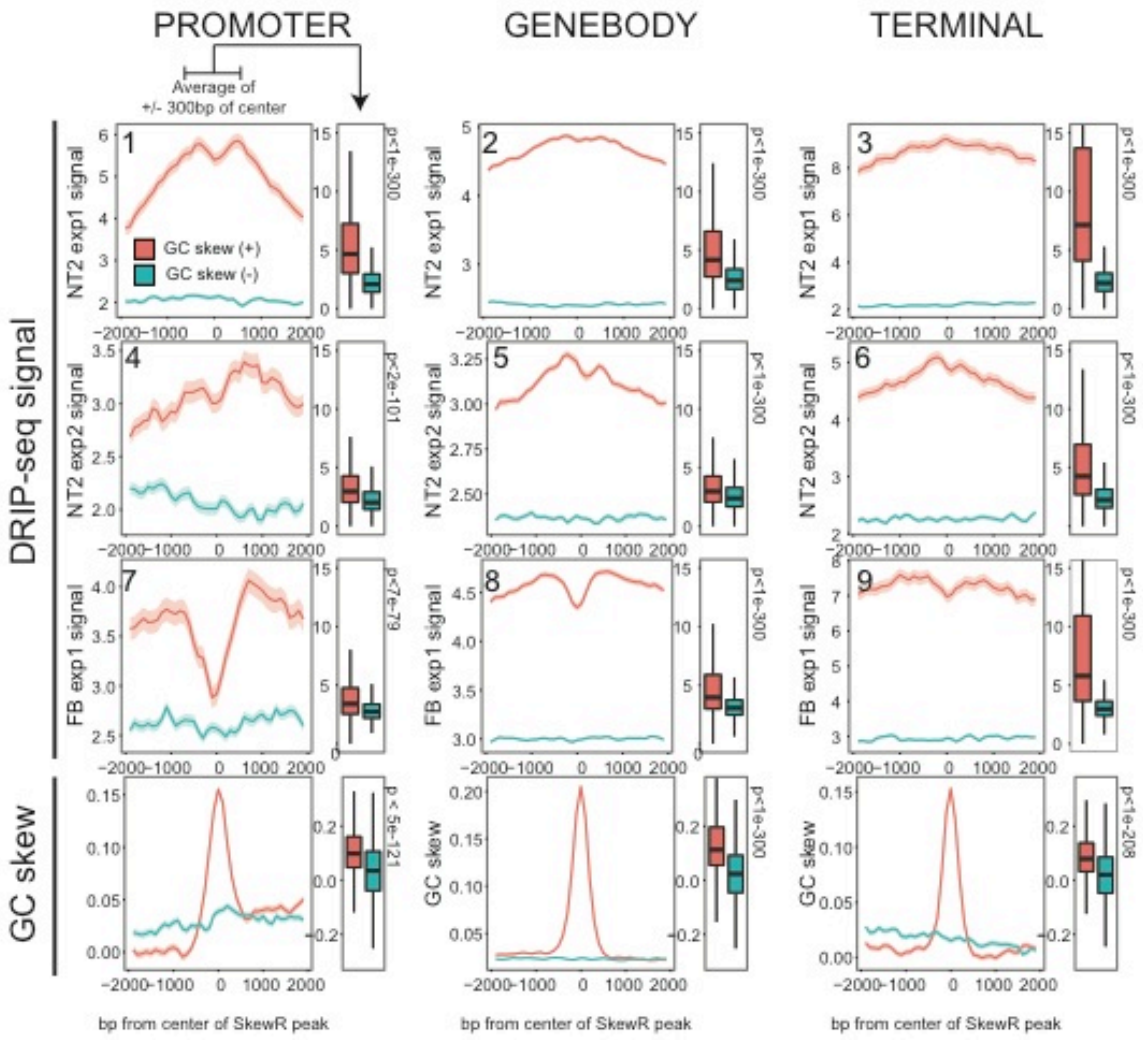

GC skew is a key determinant of DNA:RNA hybrid signal in wild type and embryonal carcinoma cells

DNA:RNA hybrid signal as measured by DRIP-seq over the three major DNA:RNA hybrid formation hotspots (promoters, gene bodies and terminators) was plotted over a +/- 2 kb window centered in the middle of annotated peaks of GC skew according to the SkewR algorithm[1]. Windows of 200bp were used for averaging, with a step size of 10 bp. In each category, the signal is broken down between loci that are GC-skewed (GC skew (+), red) and an equal number of loci that are not GC skewed (GC skew (-), blue). These loci were chosen at random and were matched for expression +/- 20%. Promoters, gene bodies, and terminal regions were analyzed separately. In each panel numbered 1-9, the DRIP-seq signal is shown as a metaplot where the line represents the median and the standard error is shaded. All three DRIP-seq experiments used in this study are shown

here. For each panel, we quantified the DNA:RNA hybrid signal over a 600bp window located at the center of the region and display the results at right under a boxplot format. The Wilcoxon Mann-Whitney test was used to determine if the values were statistically distinct between positive and negative GC skew regions. All p-values are indicated and were below 1e-50, showing strong significance. The GC skew signal itself for all loci is shown at the bottom.

Supplementary Figure 4

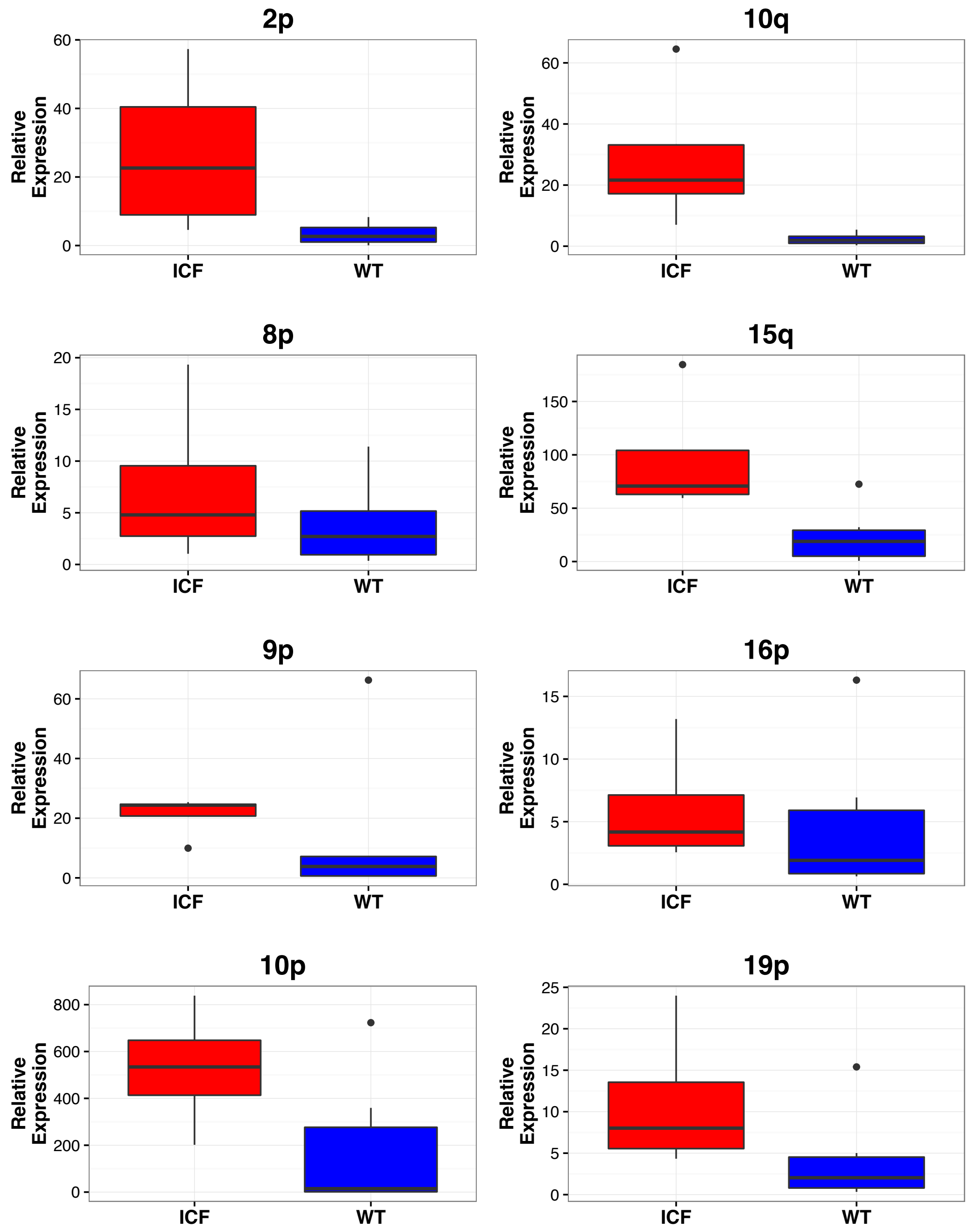

TERRA levels are elevated in ICF compared to WT LCLs

RNA was extracted from four ICF-LCLs and six WT or carrier LCLs and subjected to qRT-PCR as described in “Methods”. Box plots represent the relative TERRA expression in ICF LCLs (Red) and in WT/carrier LCLs (Blue), at eight chromosome ends (2p, 8p, 9p, 10p, 10q, 15q, 16p and 19p). Values displayed for each sample are averages of two experimental repeats. Significant differences in TERRA expression between both groups were seen at subtelomeres 2p, 10q and15q (p-value<0.05, Wilcoxon rank sum test).

Supplementary Figure 5

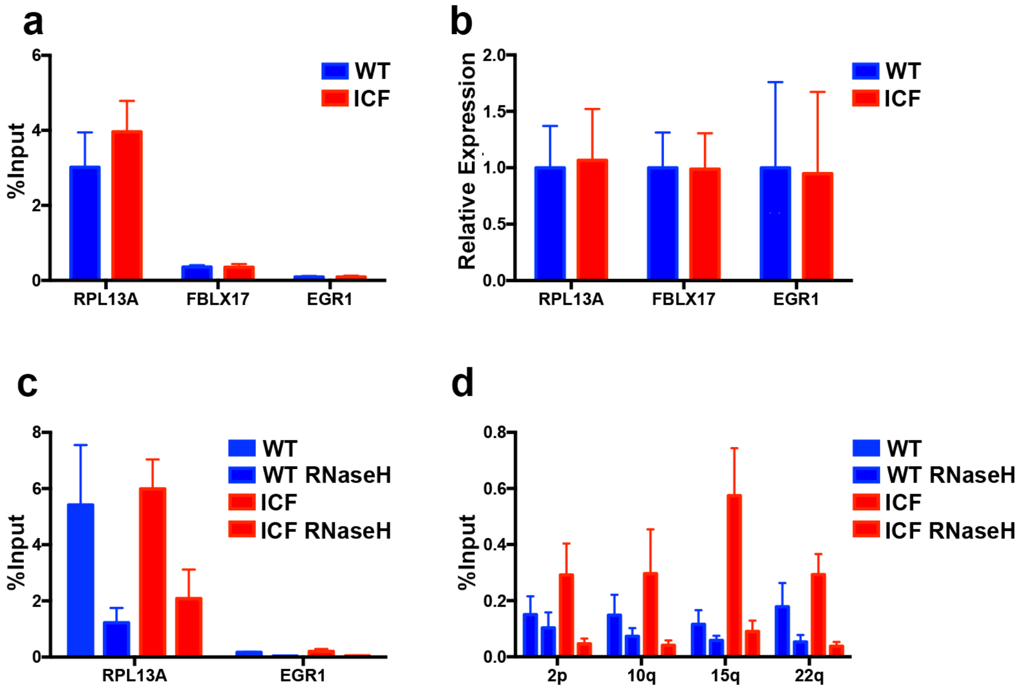

DRIP on control regions and on RNase H treated samples

(**a**) DNA extracted from five ICF and six WT LCLs was subjected to DNA:RNA immunoprecipitation (DRIP) followed by quantitative PCR analysis of three control regions. Bars and error bars represent averages and standard error of the means (SEM) for the combined data of all samples belonging to each of the groups (WT and ICF). Each of the values represents between two to five repeated experiments for each of the samples. P values obtained by Student's t test indicate that the differences between the two groups are insignificant for all control regions. (**b**) RNA extracted from four ICF LCLs (in red) and from six WT or carrier LCLs (in blue) was subjected to qRT-PCR to determine expression of RPL13A, FBLX17 & EGR1. Values displayed are averages and SEMs of each group. The difference in expression between both groups was insignificant. (**c & d**) DNA extracted for DRIP from two ICF LCLs (pG and pY, results grouped here together as "ICF") and two WT LCLs (GM8729 and GM19116c, results grouped here together as "WT") was split into two equal aliquots that were either treated (+) or untreated (-) with RNase H (M0297, *New England BioLabs*). Treatment with RNase H decreased the enrichment of DNA:RNA hybrids both in the control regions (**c**) and in the subtelomeric regions (**d**), thus serving as a control for the specificity of the S9.6 antibody for DNA: RNA hybrids. Data represents two repeats for each sample. All amplicons, with exception of EGR1 that basically demonstrates very low levels of DRIP enrichment, showed a significant decrease after treatment with RNase H (p values < 0.05, Wilcoxon signed rank test).

Supplementary Figure 6

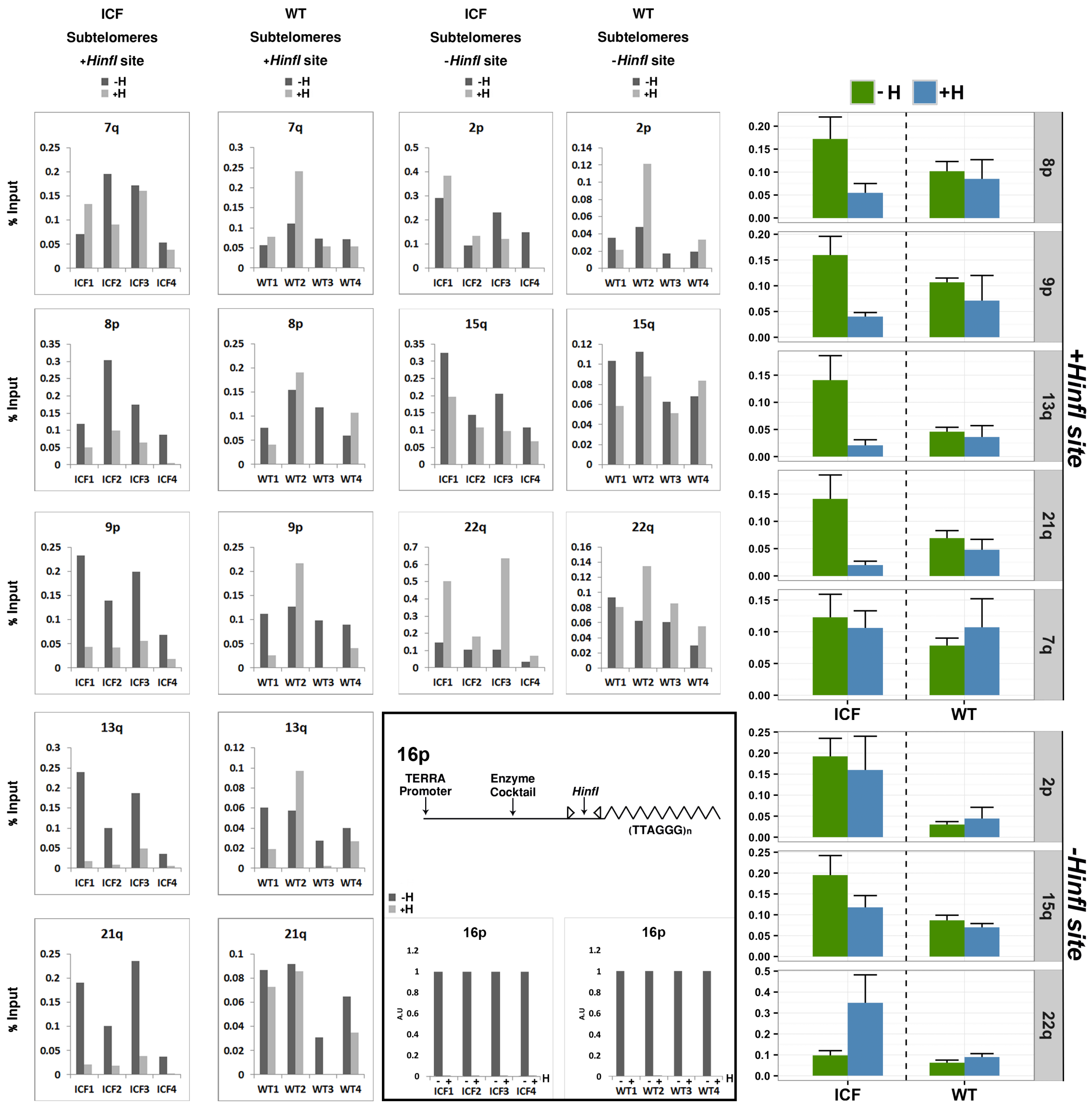

Enrichment values for DRIP performed on *HinfI*$^{+/-}$ digested DNA

DNA samples of four ICF LCLs (pCor, pG, pY and pH, designated as ICF1, ICF2, ICF3 and ICF4) and four WT LCLs (GM8729, GM19116c, 3125 and fY, designated as WT1, WT2, WT3 and WT4) were digested with the enzyme cocktail and then each sample was split into two fractions. One of the two fractions was further digested with *HinfI*, and DRIP was performed on both fractions. Presented in the gray scale graphs are the input percentage values of each sample with (+H) or without (-H) *HinfI* digestion, for several subtelomeres (the subtelomere are designated above the graph). Subtelomeres belong to

two groups – either those that contain a *HinfI* site (+*HinfI* site) or those that lack a *HinfI* site (-*HinfI* site) between the amplicon to the telomere tract, as depicted in the map in Figure 3. The colored scale graphs in the right panels present the mean and SEM of each group (ICF and WT) without (-H) and with (+H) *HinfI* digestion for each analyzed amplicon. P-values of a paired student t-test analysis of these data appear in Supplementary Table 1. In order to validate the restriction efficiency in this assay, we analyzed the input DNA of each sample with (+H) and without (-H) restriction with *HinfI* prior to DRIP, by performing qPCR with the 16p subtelomere primers. A *HinfI* restriction site is located within this amplicon (see boxed section in the figure). Following digestion with *HinfI*, no PCR amplification of this amplicon was evident.

Supplementary Figure 7

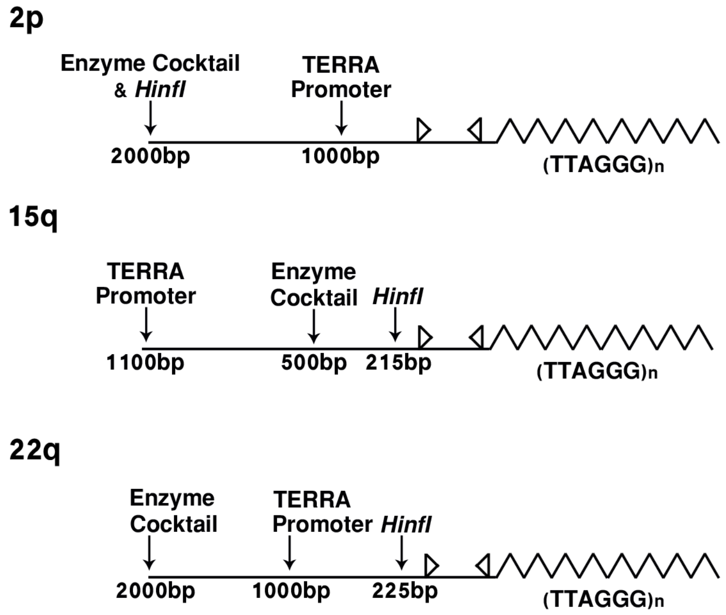

Restriction maps for the terminal regions of subtelomeres 2p, 15q, 22q
Schematic representation of a subset of subtelomeric regions subjected to DRIP-qPCR analysis following digestion with both the enzyme cocktail (*BsrGI, EcoRI, HindIII, SspI & XhoI*) and with *HinfI*. Subtelomeric sequence appears as a straight line and telomeric TTAGGG repeats as a zigzagged line. Primers used for amplifying DRIP material are depicted as head to head triangles. The positions of the TERRA promoter and the most distal digestion sites of the enzyme cocktail and of *HinfI* are depicted with arrows. The approximate distance of these regions to the telomere tract appears below the arrows.

Supplementary Figure 8

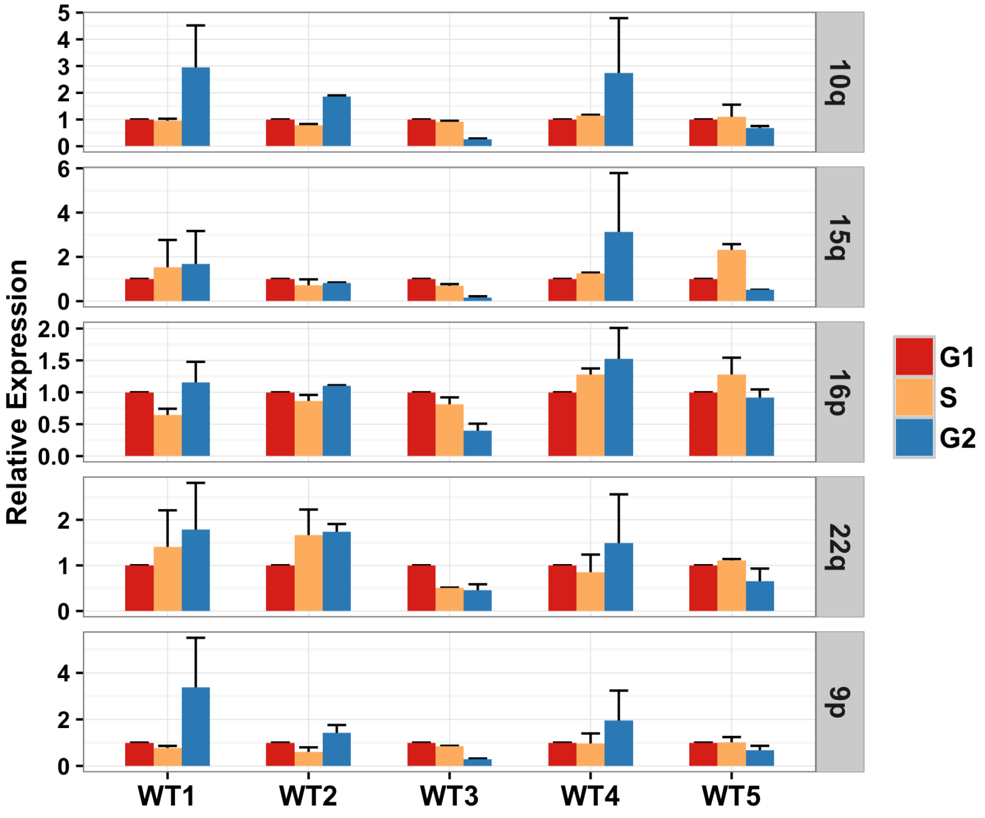

TERRA levels in WT-LCLs are relatively consistent throughout the cell cycle. Relative TERRA levels were determined in five WT LCLs (GM8729, GM19116c, 3125 fY and mY, designated as WT1, WT2, WT3, WT4 and WT5) sorted to G1, S and G2/M phases. qRT-PCR analysis of TERRA in the sorted cell cycle fractions was carried out for five telomeres (9p, 10q, 15q, 16p,and 22q). For each subtelomere, TERRA expression in the G1 sample is set at 1 and the expression levels in S and G2 phases are described relatively to G1. Bars and error bars represent means and standard error of the means (SEM) of two experimental repeats.

Supplementary Figure 9

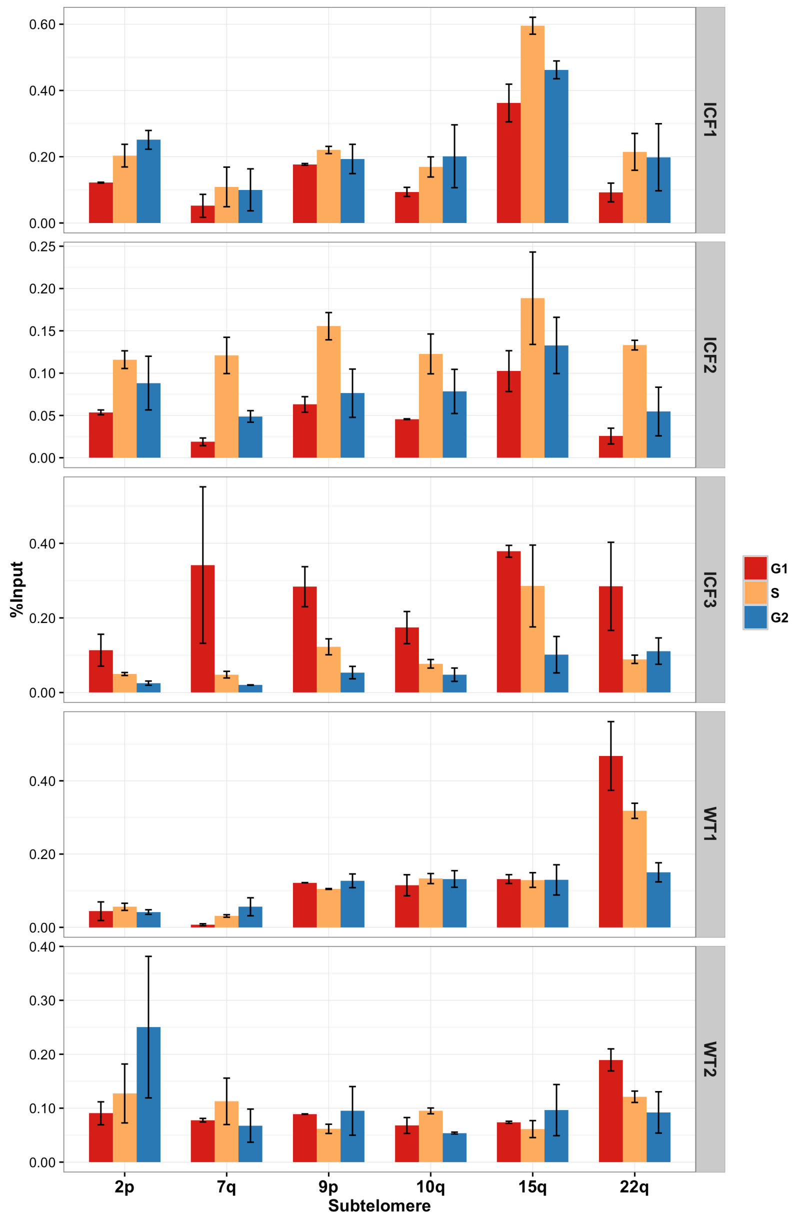

DRIP enrichment values for experiment performed on cell cycle sorted cells
Three ICF LCLs: pCor (ICF1), pG (ICF2), pY (ICF3) and two WT cells, GM8729 (WT1) and GM19116c (WT2) were sorted into G1, S and G2 phases, after which DRIP was performed and probed for the input percentage of six subtelomeric regions at the various cell cycle phases. Means and SEMs of two experimental repeats of the full input percentage data of DRIP appear in the graphs.

Supplementary Figure 10

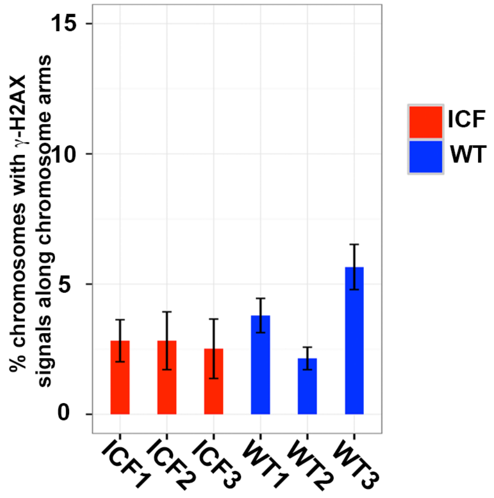

DNA damage signals along chromosome arms appear at similar frequencies in ICF and WT LCLs

Cytospun metaphase spreads of three ICF LCLs - pCor, pG and pY (designated ICF 1, 2 and 3) and three WT LCLs - GM8729, GM19116c, 3125 (designated WT 1, 2 and 3) were stained with an antibody for γ-H2AX and then scored for the percentage of γ-H2AX signals present along chromosome arms (signals at extreme chromosome-ends were not scored). At least 400 chromosomes were scored for each sample. Bars and error bars represent percentages and standard error of the means (SEM). No significant differences were consistently found when each sample was compared to all other samples in a Proportion test.

Supplementary Figure 11

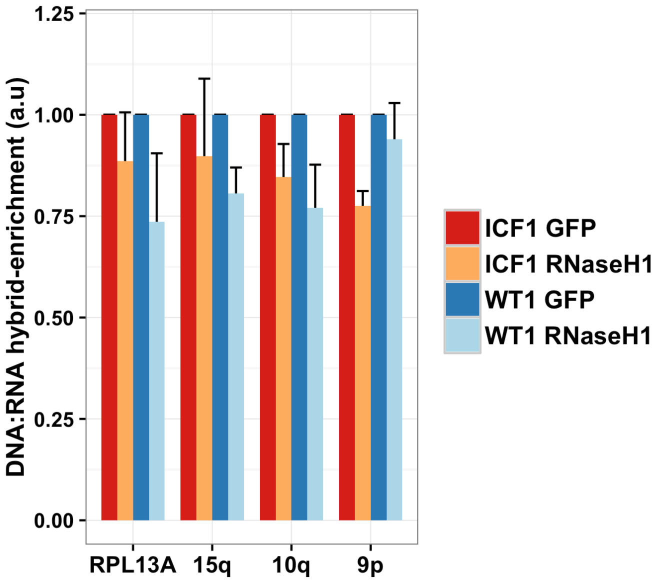

RNase H1 overexpression reduces DNA:RNA hybrids at control and telomeric regions

RNase H1-GFP (RNaseH1) or cytoplasmic GFP (GFP) were expressed in ICF1 and WT1 LCLs. Following sorting based on GFP expression, DRIP (DNA:RNA ImmunoPrecipitation) analysis was carried out for three subtelomeric regions (15q, 10q, 9p) and one control region (RPL13A). DRIP values for the GFP samples were set at 1. Bars and error bars are averages and SEM from two experiments. The DNA:RNA hybrid levels at the subtelomeric regions for each of the samples (ICF1 & WT1) significantly decreased in the samples expressing RNase H1-GFP (p-value = 0.04 for ICF1 and p-value = 0.026 for WT1, Wilcoxon signed rank test) in comparison to those expressing GFP alone.

Supplementary Table 1
P-values of the paired student t-test between the non-digested and *HinfI*-digested-samples, displayed in the colored graphs in Figure S6

	2p	7q	8p	9p	13q	15q	21q	22q
ICF	0.626	0.646	0.031	0.027	0.058	0.044	0.048	0.122
WT	0.547	0.475	0.680	0.470	0.611	0.289	0.044	0.211

Supplementary Table 2

P-values of the proportions test between samples in Figure 6c

	WT3	WT1	WT2	ICF1	ICF2	ICF3
WT3	1.0000	0.7924	0.0002	0.0007	0.0009	0.0000
WT1	0.7924	1.0000	0.0000	0.0002	0.0004	0.0000
WT2	0.0002	0.0000	1.0000	0.0000	0.0000	0.0000
ICF1	0.0007	0.0002	0.0000	1.0000	0.8198	0.0335
ICF2	0.0009	0.0004	0.0000	0.8198	1.0000	0.1228
ICF3	0.0000	0.0000	0.0000	0.0335	0.1228	1.0000

Supplementary Table 3

P-values of the proportions test between samples in Figure 6d

	WT-GFP	WT1-RNaseH	WT2-GFP	WT2-RNaseH	ICF1-GFP	ICF1-RNaseH	ICF3-GFP	ICF3-RNaseH
WT-GFP	1.0000	0.0119	0.5080	0.7353	0.0000	0.7270	0.0000	0.0006
WT1-RNaseH	0.0119	1.0000	0.0529	0.0198	0.0588	0.0447	0.0005	0.3491
WT2-GFP	0.5080	0.0529	1.0000	0.8017	0.0000	0.8953	0.0000	0.0030
WT2-RNaseH	0.7353	0.0198	0.8017	1.0000	0.0000	1.0000	0.0000	0.0008
ICF1-GFP	0.0000	0.0588	0.0000	0.0000	1.0000	0.0000	0.0799	0.5448
ICF1-RNaseH	0.7270	0.0447	0.8953	1.0000	0.0000	1.0000	0.0000	0.0031
ICF3-GFP	0.0000	0.0005	0.0000	0.0000	0.0799	0.0000	1.0000	0.0322
ICF3-RNaseH	0.0006	0.3491	0.0030	0.0008	0.5448	0.0031	0.0322	1.0000

Supplementary Table 4

Primers for RT-PCR and DRIP experiments

	**Forward primer**	**Reverse primer**	**PCR product length (bp)**	**Position of *HinfI* site**	**Primers amplify additional subtelomeres?**	**Genomic location (UCSC)**	**Promoter/TSS**
**2p**	CGCATCGACG GTGAATAAAA	GCCTAACTCGTG TCTGACTTTGAG	67	Downstream to PCR primers***	No	Chr2:10717+10839	Yes,Yes
**7q**	TTCAGACGGG CTTTTGGTTT	ATGGTGAATACA ATCCTTTCTGTTT G	135	Downstream to PCR primers***	No	Chr7:159335504-159335606	No, No
**8p**	CCGGTTGCAG CCGTTAATA	GGCTTTTGGTTTC CCGTTTT	76	Downstream to PCR primers***	No	Chr8:205881-205956	Yes,Yes
**9p**	CGGAAAACGG GAAAGCAAA	CGTTCCGACGCT GCAAGT	102	Between PCR primers**	Xq/Yq	ChrX:156029721+156029823/ chrYy:57216241+57216343/c hr9:10536-10638	Yes,Yes
**9p hinf ******	GGGCGCATTA ACGGTGAATA	CCGCACTGAACC GCTCTAAC	85	Downstream to PCR primers***	No	Chr9:10636-10720	Yes,Yes
**10p**	GCCACAGCGA CGGTAAATAA A	CCCTCACCCTTCT AACTGGACTCT	80	Between PCR primers**	18p	Chr10:10426- 10505/chr18:10622-10701	Yes,Yes
**10q**	AACCTGAACC CTAACCCTCC	ATTGCAGGGTTC AAGTGCAG	109	Upstream to PCR primers*	13q,2q 4bp difference between amplicons	Chr10:133786972- 133787080/chr13:114353851- 114353959/chr2:242183213- 242183321	Yes,Yes
**12p**	AGAACTCTGC TCCGCCTTC	GTTGCGTTCTCTT CAGCACA	102	Upstream to PCR primers*	No	Chr12:10630+10731	Yes, No

**13q**	CCCGCTTTCCA CACTAAACC	CGCATCGACAGT GAATAAAATCTT T	90	Downstream to PCR primers***	10q,21q,22q,1 q,2q,4q	Chr10:133786820-133786909/chr13:114353699-114353788/chr21:46699599-46699688/chr22:50807545-50807634/chr1:248945306-248945395/chr2:242183061-242183150/chr4:190204263-190204352	Yes,Yes
**15q**	AACCCTAACC ACATGAGCAA CG	CTCGCCTTAGCTT GGGAG	125	Upstream to PCR primers*	No	Chr15:101980649-101980774	Yes,Yes
**16p**	AACGGTTCAG TGTGGAAAAT GG	CAACTGGACCCT GCAATGC	102	Between PCR primers**	No	Chr16:10132-10233	Yes,Yes
**19p**	GCATCGACGG TGAATTAAAT CTT	GGCTTTTGGTTTC CCGTTTT	101	Between PCR primers**	No	Chr19:246147-246247	Yes,Yes
**21q**	GAATAAAATC TTTCCCGGTTG CT	CCGCTTTCCACAC TAAACCATT	77	Downstream to PCR primers***	22q/1q	Chr21:46699611+46699687/chr22:50807557+50807633/chr1:248945318+248945394	Yes,Yes
**22q**	CGAAACAGAA CCCGAAGCAG	TGCACACATGAC ACCCAAAA	131	Upstream to PCR primers*	21q/1q one bp difference between amplicons	Chr21:46699569-46699688/chr22:50807515-50807634/chr1:248945276-248945395	Yes,Yes

* *HinfI* digestion does not release telomeric repeat

** *HinfI* digestion abolishes PCR amplification - control for digestion

*** *HinfI* digestion releases telomeric repeats from subtelomere

**** A different primer set was used for subtelomere 9p in the *HinfI* experiment because a *HinfI* restriction site occurs between the 9p primers.

References

1 Ginno, P. A., Lott, P. L., Christensen, H. C., Korf, I. & Chedin, F. R-loop formation is a distinctive characteristic of unmethylated human CpG island promoters. *Mol. Cell* **45**, 814-825 (2012).

2. Arora, R., Lee, Y., Wischnewski, H., Brun, C.B., Schwarz, T & Azzalin, C.M. RNaseH1 regulates TERRA-telomeric DNA hybrids and telomere maintenance in ALT tumour cells. *Nature Comm.* 5, 5220, doi:10.1038/ncomms6220 (2014).
